# Phase I study of OM-174, a lipid A analogue, with assessment of immunological response, in patients with refractory solid tumors

**DOI:** 10.1186/1471-2407-13-172

**Published:** 2013-04-02

**Authors:** Nicolas Isambert, Pierre Fumoleau, Catherine Paul, Christophe Ferrand, Sylvie Zanetta, Jacques Bauer, Kevin Ragot, Gérard Lizard, Jean-François Jeannin, Marc Bardou

**Affiliations:** 1 INSERM CIC-P803, CHU de Dijon, Batiment du Pr Marion, 14 rue Gaffarel, BP77908, 21079, Dijon Cedex, France; 2 Centre G-F Leclerc, 1 rue du Pr Marion, BP77980, 21079, Dijon Cedex, France; 3 Université de Bourgogne, Faculté de Médecine, 21000, Dijon, France; 4 EPHE, Laboratoire d’Immunologie et Immunothérapie des Cancers, 21000, Dijon, France; 5 Centre de Recherche INSERM U866, Université de Bourgogne, 21000, Dijon, France; 6 OM-Pharma, 1217, Geneva, Switzerland; 7 Service de Pharmacologie, 21000, CHU Dijon, France

**Keywords:** Immune response, OM-174, Lipid A analogue, Refractory solid tumors, Phase I

## Abstract

**Background:**

Lipids A, the lipophilic partial structure of lipopolysaccharides, induce regression of several tumor types in animal models. Rather than exerting direct cytotoxic effect, these compounds trigger the immune system which in turn stimulates secretion of cytokines, and activates the inducible nitric oxide synthase, as well as immune cell infiltration of tumors. OM-174 is an analogue of lipid A with dual action on Toll-like receptors 2 and 4. In an experimental model of peritoneal carcinomatosis induced in BDIX rats by intraperitoneal injection of syngeneic PROb colon cancer cells, it induced a complete regression of tumors. The present phase I trial was conducted to determine the maximum tolerated dose, the recommended phase II dose and biological response associated with OM-174 administered as intravenous infusion.

**Methods:**

Patients received OM-174 twice weekly for a total of 5, 10 or 15 injections of either 600, 800 or 1000 μg/m^2^. Blood samples for pharmacokinetic analysis and cytokine dosages were collected. NK cells activity and Toll-like receptors 4 polymorphism analysis were also performed.

**Results:**

Seventeen patients were included. The highest dose administered was 1000 μg/m^2^ repeated in 15 injections. The most common toxicities were a chills, fever, nausea/vomiting, diarrhea, fatigue and headache. No patient experienced haematological side effects. As no dose limiting toxicity was observed, despite a grade 3 respiratory complication, the maximal tolerated dose and recommended dose were not established. Three patients exhibited disease stabilization with a mean duration of 4 months. Pharmacokinetic profile of OM-174 was characterized by a low distribution volume and clearance. Analysis of TLR 4 polymorphysm showed that most (16/17) patients carried the wild type alleles. A progressive increase in NK cell number and activity was observed only in patients receiving 1000 μg/m^2^ of OM-174. A peak of IL-8 and IL-10 concentrations were observed after each OM-174 injection. Peaks of TNF-alpha and IL-6 concentrations were detected after the first infusion and decreased progressively suggesting tolerance.

**Conclusion:**

OM-174 therapy was well tolerated at biologically active concentrations. Whereas the recommended dose was not determined, further studies are planned in combination with chemotherapy as animal models suggest a strong synergistic antitumor effect.

**Trial registration:**

NCT01800812 (ClinicalTrials.gov Identifier).

## Background

Despite a downward trend of mortality rates [1-5], cancer remains the second cause of death, after cardiovascular diseases, in industrialized countries accounting for 570,000 deaths in the USA in 2010 [6,7].

Whereas the primary tumor can, in most cases, be efficiently treated by a combined therapeutic approach, preventing the metastatic spread of the disease is often not effective. The eradication of disseminated tumor cells present in the blood circulation and micro-metastases in distant organs therefore represents another promising approach of cancer immunotherapy. The immune system could recognize tumor-specific antigens, and eliminate cancer cells. Furthermore it has been highlighted that some cancers, such as colorectal cancer, cause direct inhibition of the host's immune response with a detrimental effect upon prognosis suggesting that stimulation of the immune system might offer a therapeutic strategy to counteract these effects [8]. This has been observed with Toll-like receptor 2 (TLR2), TLR4 and TLR9 agonists, such as BCG and paclitaxel, which have demonstrated significant benefit for the treatment of bladder and breast cancers [9-11].

Toll-like receptors (TLRs) are important sensors of foreign microbial components as well as products of damaged or inflamed self-tissues. TLRs are a family of type I transmembrane proteins that are the major pattern recognition receptors, and using TLR proteins, host can recognize the conserved molecular structures found in pathogens called pathogen-associated molecular patterns (PAMPs). In addition to their recognition of PAMPs, several TLRs have also been shown to recognize endogenous ligands associated with inflammation, which have been termed danger-associated molecular patterns (DAMPs). Then, TLRs have an important role in maintaining tissue homeostasis by regulating the inflammatory and tissue repair in responses to injury. There are 13 TLRs described in mammals with a broad expression profile but TLR11, 12 and 13 are not expressed in the human genome. TLR1, TLR2, TLR4, TLR5 and TLR6 reside in the plasma membrane and recognize extracellular ligands [12]. It is now well recognized that TLR4 is signalling receptor for lipopolysaccharides (LPS) and more specifically lipid A [13,14].

LPS are major cell wall components of Gram-negative bacteria and were found to be able to induce regression of several tumor types in animal models [15]. This antitumor activity is related to lipids A, a component of LPS with oligosaccharide core and polysaccharide chain [16-19]. Lipids A do not induce direct cytotoxicity on tumor cells but trigger the immune system [16,19] stimulating expression and secretion of several cytokines [20,21], and activating the inducible nitric oxide synthase (NOSII) [22]. They also induce activation of various immune cells which infiltrate the tumors [15,17,23,24].

OM-174 is an analogue of lipid A that exerts, in experimental models, anti-tumoral effect against different tumor types including colon and breast cancers and melanoma [17,25]. In a model of peritoneal carcinomatosis induced in BDIX rats by intraperitoneal injection of syngeneic PROb colon cancer cells, intravenous (IV) administration of OM-174 (1 mg/kg, 15 times every third day), induced complete regression of tumors and hemorrhagic ascitis in 95% of cases [17]. This anti tumoral activity was associated with cytokine secretion and NOSII activation [22].

Based on these experimental data showing that several administrations are needed to achieve antitumor effect, as well as on the overall favourable safety profile of lipid A analogues observed in phase I studies [26-28] including two with single IV-administration of OM-174, one conducted in healthy volunteers and the other in patients with solid cancer, we designed a phase I study with repeated dose of OM-174. The aim of this study was to determine tolerability, toxicities, the maximum tolerated dose (MTD) based upon predetermined dose limiting toxicities (DLT) and the recommended phase II dose of OM-174 administered as a 15 minutes intravenous infusion, repeated twice weekly for 5, 10 or 15 administrations.

This new phase one was deemed necessary as animal data suggested that repeated administration of OM-174 were needed to observe anti-tumor effect, with a maximal effect observed after 15 injections. This study also aimed to assess the pharmacokinetic profile and the biological response induced by OM-174 in the multidose administration schedule.

## Methods

This was a single center phase I study conducted in the clinical research unit (Centre Georges-François Leclerc - INSERM CIC-P 803) in Dijon France. The study protocol and its amendments were approved by Ethics Committee and Health Authorities (NCT01800812 (ClinicalTrials.gov Identifier)). The trial was designed according to the current Declaration of Helsinki [29] and conducted in accordance with Good Clinical Practice Guidelines. Written informed consent was obtained from each participating patient before study entry. The study was supported by a grant from the French Ministry of health (Projet Hospitalier de Recherche Clinique, PHRC 2002). OM-174 was a generous gift from OM-Pharma (Geneva, Switzerland).

### Patients selection

Patients were eligible if they were ≥ 18 years, had histologically proven metastatic solid tumor with measurable progressive disease outside the central nervous system for which no standard curative measures exist. Other requirements included predicted life expectancy ≥ 3 months; a WHO performance status (PS) ≤ 2, no severe or uncontrolled medical conditions; adequate hematologic count), no severe liver impairment and adequate renal function according to international standard values.

Patients must have recovered from adverse events due to agents administered > 4 weeks earlier or ≥ 6 weeks for nitro urea or mitomycine C.

Main non-inclusion criteria were medical history of cardiac disorders; medical history of auto immune disease and corticosteroids use.

### Study design and dose escalation scheme

Based on animal models suggesting that maximal anti-tumoral was achieved with 15 infusions it was decided to include the number (Nb) of infusions in the definition of Dose Levels to confirm the good tolerance of a growing number of OM-174 injections. A standard phase I design was thus used with 3 to 6 patients to be included per each OM-174 infusion dose level (DL: 600, 800 and 1000 μg/m2) and each number of infusions (5, 10, 15). Patients were observed for the first 2 weeks before the next dose escalation was deemed possible. The MTD was defined as the DL at which two or more out of 3 or 6 patients of a DL developed a dose-limiting toxicity (DLT). The recommended dose (RD) for further clinical testing, defined as the DL below the MTD, had to be assessed in at least 6 patients.

### Dose Limiting Toxicities (DLTs)

DLTs were defined using the Common Terminology Criteria for Adverse Events version 2.0 (CTCAE) as any of the following events deemed attributable to OM-174, i.e. occurring during the time of the deliverance of this drug and a follow-up period of 30 days: Grade 4 neutropenia lasting 5 or more consecutive days; febrile neutropenia; neutropenic infection; Grade 4 thrombocytopenia or Grade 3 thrombocytopenia with bleeding requiring platelet transfusion; Grade 4 anaemia, any Grade 3 or greater drug related non haematological toxicity (except nausea, vomiting, alopecia).

### Study drug administration

In this open-label, nonrandomised phase I study, with double dose escalation protocol, OM-174 was administered intravenously as a 15 minutes infusion twice weekly at repeated intervals of 3 and 4 days. OM-174 was manufactured by OM Pharma Pharmaceuticals, Inc (Geneva, Switzerland).

Based on the results of a previous unpublished phase I study of single administration of OM-174 to cancer patients where only one serious adverse event, a severe transient hypoxia, occurred at the dose level of 800 μg/m^2^, with no other safety concerns with OM-174 dose up to 1300 μg/m^2^, it was decided that all patients would have to receive a first administration of 600 μg/m^2^ to reduce the risk of severe reaction. The dose escalation protocol and the dose levels are depicted in Table 1.

**Table 1:** Dose escalation schedule

**Dose level**	**Nb of infusion per patients**	**Dose per infusion**	**Nb of patients included**	**Total of infusions delivered**
1	5	600 μg/m^2^	3	15
2	10	600 μg/m^2^	3	30
3	15	600 μg/m^2^	3	36
4	5	800 μg/m^2^	3	15
5	10	800 μg/m^2^	3	17
6	15	800 μg/m^2^	0	0
7	5	1000 μg/m^2^	0	0
8	10	1000 μg/m^2^	2	25
9	15	1000 μg/m^2^	0	0

Suppression of dose level 6 and 7 after amendment of the protocol.

### Pre-treatment evaluation and follow up

At baseline, physical examination and routine laboratory tests were performed and WHO PS determined. Complete blood cell count was obtained on a weekly basis. Biochemical profile was performed at inclusion and each drug administration. All toxicities were graded using the CTCAE version 2.0. Each patient had to receive the full course of treatment, except in case of early progression, unacceptable toxicity, serious intercurrent illness, or patient refusal. Tumor measurements were performed at baseline, at the end of the treatment (i.e 3, 5 and 8 weeks after the beginning of treatment in the cohorts with 5, 10 and 15 infusions respectively). In case of control of disease, tumor assessment was performed on a monthly basis. Responses were assessed using RECIST [30].

### Pharmacokinetic assessment

Blood samples for pharmacokinetic (PK) of OM-174 were drawn at pre-dose, 1 h, 2 h, 4 h, 24 h for the first infusion and at pre-dose, 1 and 2 h for the 2^nd^, 3^rd^, 5^th^, 9^th^ and 15^th^ infusions, when appropriate. For all other interim injections, two blood samples were collected, before and at the end of IV-infusion.

PK parameters assessed were Cmax, Tmax, area under the curve (AUC) Last, and AUC0-inf., systemic clearance (Cl), volume of distribution (Vdβ) and elimination half-life (t1/2_lambda_z).

### Cytokines measurement

Blood samples were collected for cytokine dosage (IL-1β, IL-6, IL-8, IL-10, IL-12, TNF-α) at the same time as samples for pharmacokinetic analysis. Cytokine measurements were determined by Cytometric Bead Array (CBA; BD Biosciences). Serum of patients were incubated with labeled capture beads and detection reagent for 3 h in the dark at room temperature and were analyzed with a flow cytometer (FACSCalibur; BD Biosciences) using the respective CBA Analysis software (BD Biosciences) and Bender MedSystems software. Each experiment was performed in duplicate to ensure for reproducibility of results, as previously described [31,32].

### Natural Killer activity analysis

Blood samples for NK cell activity analysis were collected before and 2 h after the first and the 2^nd^, 3^rd^, 9^th^ and 15^th^ injections. NK cells were isolated with lymphocytes from blood by density gradient centrifugation (Unicep, Novamed, Jerusalem) and purified with CD56 coated magnetic microbeads (Miltenyi Biotec). They were incubated with or without recombinant IL-2 for 40 h, then incubated in 96-well plates for 4 h with K562 human leukemia cells or Daudi cells which were used as NK and LAK target cells (TC), to yield a NK :TC ratio of 10. NK cytotoxicity was classically calculated as in chrome test assay.

### TLR 4 polymorphism analysis

The analysis of 2 single nucleotide polymorphisms of TLR TLR4_D299G_ and TLR4_T399I_ was performed as previously described by Ducloux et al. [33].

Prior to polymorphism analysis, gDNA was extracted from serum and amplified using the Whole Genome Amplification method Polymorphisms analysis was then performed using restriction fragment length polymorphism method and direct PCR sequencing for TLR4 (2 polymorphisms).

### Statistical analysis

Because of the nature of this study, no formal statistical analysis was planned. Evaluation of the data consisted primarily on summary displays (i.e., descriptive statistics and graphs). Qualitative data were summarized by frequency and percentages, while quantitative data were summarized by descriptive statistics.

## Results

### Patient characteristics

From November 2004 to July 2007, 17 patients were enrolled. Their characteristics are described in Table 2. Main tumour type was colon or rectal cancers, followed by breast cancer and ovarian cancer. All patients were included after failure of optimal standard treatment, including chemotherapy which consisted in the majority (58.8%) of cases in 4 or more lines, according to their tumor type.

**Table 2:** Patient’s characteristics

**Characteristic**	**Nb of patient (%)**
Nb of registered and treated patients	17
Age (years)	
Median	59
Range	44 - 75
Gender	
Male	9 (53)
Female	8 (47)
WHO performance status	
0	3 (18%)
1	12 (70%)
2	2 (12%)
Type of cancer	
Colon or colorectal cancer	7 (41%)
Breast cancer	5 (29%)
Ovarian cancer	2 (12%)
Non-small-cell-lung cancer	1 (6%)
Head and neck cancer	1 (6%)
Hepatocarcinoma	1 (6%)
Status disease	
Minimal	5 (29%)
Bulky	12 (71%)
Metastatic sites	
Liver	11 (65%)
Lung	9 (53%)
Lymph nodes	5 (29%)
Bone	3 (18%)
Skin	3 (18%)
Brain	1 (6%)
Adrenal gland	1 (6%)
Previous lines of chemotherapy	
Median	5
Range	1 - 11
Distance by last chemotherapy	
Median (months)	1,5
Range	1 - 18

### Drug delivery

A total of 138 infusions were administered with a median number of 5 injections (range: 3 to 15). Protocol was amended after 15 patients have been included, to suppress dose level 6 and 7 to facilitate patient enrollment. The investigator had the choice to continue treatment as up to a total of 15 infusions if a benefit was observed for the patient.

Number of patients and total of delivered infusions in each DLs are depicted in Table 1.

The majority of the subjects (82%) received all the administrations planed in each DL. At DL3 (15 injections of 600 μg/m^2^), a 63-year-old woman with a metastatic breast cancer, received only 6 infusions due to a rapid alteration of her general status in relation to a progressive disease; at DL 5 (10 injections of 800 μg/m^2^), a 46-year-old woman with ovarian cancer and a 68-year-old man with liver cancer received respectively only 4 and 3 out of 10 OM-174 infusions because of a progressive disease in the first case and a severe adverse event, bronchospasm, in the other one.

### Safety results

All patients were evaluable for safety analysis. No haematological disorders were observed after OM-174 administration, in any patient.

Non haematological adverse events are shown in Table 3, according to treatment cycle. The most frequent adverse event was flu-like syndrome, reported by 12 of the 17 patients. However no grade 3 and 4 events were observed. Chills occurred in 12 patients, associated with fever in 7 cases. For patient’s convenience, IV acetaminophen had to be administered after 68 of 138 (49%) OM-174 infusions and Non-Steroidal Anti-Inflammatory Drugs (NSAIDs) after 19 (14%) OM-174 infusions to treat flu-like syndromes.

**Table 3:** Incidence of worst grade for most common drug-related non haematological adverse events per cycle

	**NCI CTC grading**		
**Adverse Events**	**All grades N (%)**	**G3 N (%)**	**G4 N (%)**
Chills	12 (70)	-	-
Fever	7 (41)	-	-
Nausea	4 (23)	-	-
Vomiting	4 (23)	-	-
Fatigue	4 (23)	-	-
Headache	3 (17)	-	-
Diarrhoea	3 (17)	-	-
High blood pressure	1 (7)	-	-
Dizziness	1 (7)	-	-
Somnolence	1 (7)	-	-
Dysphagia	1 (7)	-	-
Cholestasis	1 (7)	-	-
Cytolysis	1 (7)	-	-
bronchospasm	1 (7)	-	-

Gastro-intestinal disorders were seen in 7 patients. Four of them had nausea or vomiting and 3 diarrhea. Four patients experienced fatigue and three reported having headache after OM-174 administration.

All other adverse events, hypertension not requiring treatment, dizziness, somnolence, and dysphagia, were graded one and were experienced by a single patient.

Grade 1 hepatic cytolysis and cholestasis were observed in a patient who had a progressive disease, and it was concluded that this abnormalities were unlikely related to the drug.

No death related to the drug was observed during the study.

### Determination of the MTD

All the 17 patients enrolled were evaluable for the determination of the MTD, but since no DLT was observed its determination was not feasible.

However, a patient included in the study for progressive hepatocarcinoma and scheduled to be treated at dose level 5 (10 infusions of 800 μg/m^2^ of OM-174) experienced a serious adverse event after the third administration. The patient exhibited a severe bronchospasm with oxygen desaturation, hypoxemia immediately at the end of the treatment. He fully recovered after an IV-infusion of corticosteroids but did not receive any further administration of the study drug. This was not considered as a dose-limiting toxicity since it was not related to the dose and was more likely to be of immunological origin, as patients receiving the next dose of 1000 μg/m^2^ and the 5 additional patients who received the same dose of OM-174 (800 μg/m^2^) for at least five administrations did not experience such grade 3 or 4 toxicity.

### Pharmacokinetics

All 17 patients were evaluable for PK.

After the first IV infusion at the single dose of 600 μg/m^2^, OM-174 pharmacokinetic profile showed a Tmax longer than the end of the infusion, a Cmax reaching 394 ng/ml and a two phases elimination process (an initial phase of only a few minutes and a second phase lasting about 22 hr). Clearance and volume of distribution were low, 0.11 L/h (66 mL/hr/m^2^) and 4.6 L (2643 mL/m^2^), respectively. No accumulation occurred at the multiple dosages of 600 μg/m^2^ twice a week, whereas a small accumulation was evidenced for the higher 800 and 1000 μg/m^2^ dosages.

### Cytokines dosage

**Figure 1:**
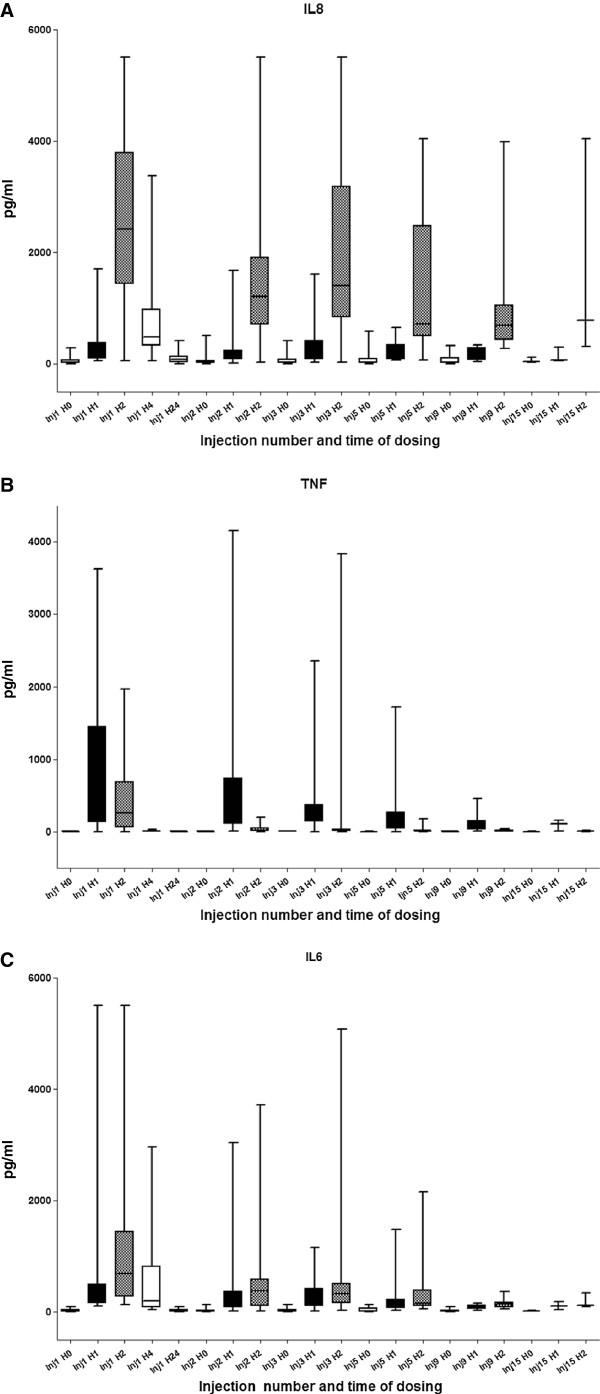
**a Increase of IL-8 concentration after each injection of OM-174 with a peak at second hour, independently of dose of OM-174 and number of injections.** Black, doted and white boxes represent the value of the cytokine of interest 1, 2 and 4 hours after the injection of interest. Analyses were conducted on all 17 patients according to their number of OM-174 injections. **1b** and **1c** Both concentration of TNF-α and IL-6 decreased after each injection of OM-174 suggesting a tolerance. The peak of secretion is observed respectively 1 and 2 hours after the first injection of OM-174 for TNF-α and IL-6. Black, doted and white boxes represent the value of the cytokine of interest 1, 2 and 4 hours after the injection of interest. Analyses were conducted on all 17 patients according to their number of OM-174 injections.

All patients were evaluable for this analysis. Cytokine changes induced by OM-174 infusions are summarized in Table 4. Several profiles were observed. Unlike IL-1β and IL-12 which were not detectable (data not shown), IL-8 (Figure 1a) and to a lesser extend IL-10 increased after each OM-174 injection within same range, whereas peaks of TNF-α and IL-6 concentrations decreased from the first to the last injection of OM-174, suggesting tolerance (Figures 1b and 1c).

**Table 4:** Results of dosage of cytokines after the administration of OM-174

		**Dose level in ug/m**^**2**^	**Median pg/ml**	**Minimum pg/ml**	**Maximum pg/ml**
**IL8**	**2h**	600	2417	54	5000
		800	3053	443	5000
		1000	1469	1169	1768
**IL6**	**2h**	600	461	3	1521
		800	1403	150	5000
		1000	676	296	1058
**IL10**	**2h**	600	42	4	138
		800	90	4	326
		1000	48	43	52
**TNF**-α	**1h**	600	907	4	1446
		800	684	4	1970
		1000	754	485	1024
**IL12**	**2h**	600	12	0	35
		800	0	0	11
		1000	8	8	9
**IL1**β	**1h**	600	34	0	115
		800	0	0	0
		1000	13	12	14

### Natural killer activity analysis

**Figure 2:**
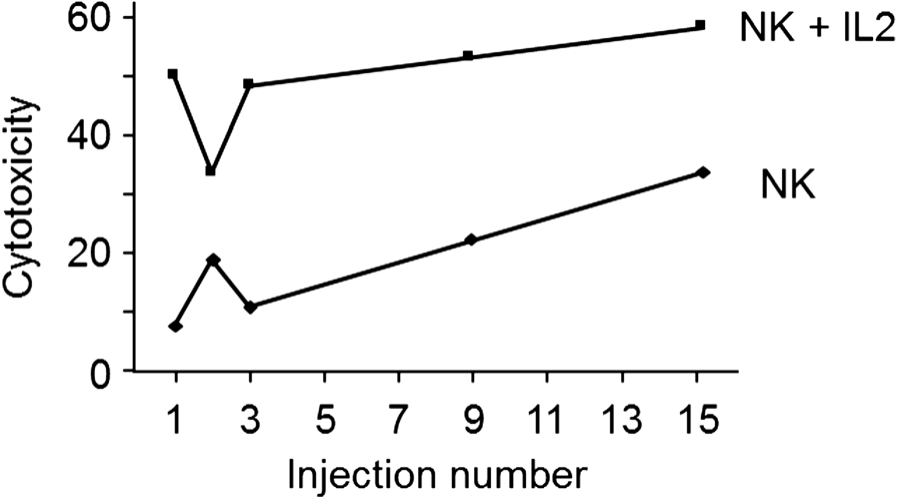
Increase of NK activity after each injection of 1000 μg/m^2^ OM-174 and in association with IL-2.

When all patients with available data were considered, neither the number nor the activity of NK cells were increased. Nevertheless in patients receiving 15 injections of OM-174, irrespective of the dose, NK cell number was increased compared with baseline value, from 0.67 ± 0.25 × 106 to 2.21 ± 0.17 × 106 NK cells (p<0.005). Furthermore, patient 17, who received the highest dose of OM-174 (1000 μg/m^2^) and the highest number of injections (15) showed a significant progressive increase in NK cell activity as OM-174 infusions were repeated (Figure 2).

### TRL 4 polymorphism analysis

All but one (16 out of 17) patients of this cohort carried the wild type alleles for TLR4_D299G_ and all for TLR4_T399I_ polymorphisms, making us unable to assess the relationship between TLR4 polymorphisms and response to OM-174, either in terms of biological or clinical response.

### Antitumor activity

Responses were assessed by the investigator in every patient.

One patient with hepatocellular carcinoma who was withdrawn from the study after the third administration because of grade 3 toxicity (bronchospam) was not available for tumor response.

Stabilization of disease was documented in 3 patients (17%) and seen at DL 3, DL4 and DL8. Characteristics of these patients are summarized in Table 5. Although no formal statistics were performed, no trend was observed for a correlation between disease course and any of the biological parameters that were assessed in the present study.

**Table 5:** Characteristics of patients with stable disease

**Nb of patients**	**Age (YEARS)**	**Sex**	**Previous lines of chemotherapy**	**DL**	**Type of cancer**	**Duration of stable disease (MONTHS)**	**Global survival (MONTHS)**
Patient 9	60	Male	11	3	rectum	1	6
Patient 12	62	Male	3	4	colon	9	19
Patient 17	57	Male	4	8	lung	2	12

## Discussion

Our study strengthens data on the overall very favourable safety profile of OM-174, as administered for up to 15 injections as a biweekly infusion and up to 1000 μg/m^2^ which is the recommended dose. Chills (70%) and fever (41%) were the most common adverse advents, with no grade 3 or 4, explaining that the MTD could not be determined, and were reversible with acetaminophen, NSAID or steroid treatments. Furthermore no increase in severity occurred as injections were repeated. It might be argued that giving acetaminophen or NSAIDs to patients with grade 1 or 2 fever might impair antitumoral effect as it has been suggested that response to IL-2 plus interferon-alpha might be altered by acetaminophen in patients with metastatic melanoma [34]. Nevertheless our patients were included prior to this report that has never been published as a full paper and only a trend was suggested. The number of patients with disease stabilization observed in our study (3) is too small to allow for an appropriate assessment of the influence of acetaminophen or NSAIDs use in tumour response, but it has to be noticed that these 3 patients all received acetaminophen.

Others adverse events included nauseas, vomiting, fatigue, headache and diarrhoea and were generally of mild to moderate intensity. The more severe adverse event was an episode of bronchospasm which occurred for a patient with a progressive hepatocarcinoma after the third infusion of 800 μg/m^2^.

The safety profile of OM-174 we describe here is similar to that observed in others studies with other lipids A [26-28], whether the lipid A was of synthetic origin as SDZ MRL 953 [26] and ONO-4007 [27], or prepared from S typhimurium and minnnesota as in the phase I trial performed by Vosika et al. Similarly to our study, Vosika et al. described bronchospasm in one patient, considered as a severe adverse event. In this study another patient experienced, at the highest dose level (500 μg/m^2^) a severe hypotension which was considered by the authors as a DLT. It has to be noted that we assessed twice higher dose and number of injections (15 vs. 8) of lipid A than in the study by Vosika et al [28].

OM-174 PK parameters appeared to be independent of dose within the dose range of 600 – 1000 μg/m^2^. A trend toward higher OM-174 concentrations was observed when administered doses increased, but the difference was not significant. OM-174 is a drug with a low systemic clearance (0.11 L/h) and a small distribution volume (4.6 L). Our results are in agreement with those of de Bono et al. [27] who described that the pharmacokinetic of ONO-4007, another synthetic lipid A analogue, appeared to be independent of dose and showed linearity with respect to time. ONO-4007 was described to have a low systemic clearance (approximately 1.3 ml/min) and a small volume of distribution (5-8 liters) with a long t1/2 of 74-95 h. These data validated the twice weekly administration protocol. It has previously been reported that shorter intervals between repeated injection of LPS lead to the development of LPS tolerance [35] and similar tolerance might have occurred with Lipid A analogues even if it has never been studied.

Pharmacodynamic studies showed OM-174 IV-infusion induced a significant increase in IL-8, IL-10, IL-6 and TNF-α concentrations. IL-8 and IL-10 concentrations increased after each OM-174 injection whereas TNF-α and IL-6 concentrations were highest after the first OM-174 injection and declined thereafter as OM-174 injections were repeated. This response was not correlated to the dose of OM-174 and is known as tolerance. The occurrence of tolerance or host desensitization in animals and humans administered multiple doses of microbial products has long been recognized [36]. It was reported with OM-174 in preclinical studies. In the study by De Bono et al. [27], an increase in IL-1α, IL-6, and TNF-α concentration was observed whereas it was not the case with SDZ MRL 953 in the study by Kiani et al. [26]. Our study is in partial agreement with both studies as we observed an increase in TNF-α, IL-6, IL-8 and IL-10, but not in IL-1β or IL-12. This increase of secretion of TNF-α is in the same magnitude as that observed with ONO-4007 (1970 versus 1200 pg/ml), suggesting a similar biological activity and confirming that OM-174 activates the TLR4 receptor as has been demonstrated in preclinical models. The variations of cytokines secretion could be due to the dose (from 2 to 125 mg/patient), or administration schedules (every day for SDZ MRL 953, every week for ONO-4007) differences, between the three clinical assays. However, the best clinical tolerance of OM-174 makes it a drug of choice for clinical development.

Interestingly, our study also suggests that a sufficient number of OM-174 injections has to be administered to reach a biological activity since a trend toward an increase in NK cells number and activity was observed only in patients receiving 15 infusions. As only three patients received 15 infusions these findings deserve further studies to be confirmed.

As OM-174 has been shown, in mice, to induce TNF-α production via TLR4 receptors, a family of receptors which recognized pathogen-associated microbial structure and are involved in innate immune cell activation and antitumor immune response inducing tumor cell death [37], we search for possible mutations that may explain inter patient variability in terms of biological and clinical activities. Sixteen out of seventeen patients in our study carried the wild type suggesting that TLR4 responsiveness was unaltered in our population. Since no functional analysis was performed, we cannot formally rule-out a TLR4-mediated variability in clinical response, but in the absence of genomic variability this is unlikely to be the case. However, the finding of TNF secretion after administration of OM-174 in our study as has been observed in mice suggested that OM-174 activates TLR4 well.

We did not observed objective tumour responses, as it was the case in the Vosika [28] or de Bono [27] studies, but disease stabilisation occurred in 3 patients including 2 patients with colo-rectal carcinoma and one patient with non-small cell lung carcinoma. As disease stabilisation was observed at 3 different dose levels including three different doses and number of infusion, and as no patients were included in some dose levels, no trend can be suggested toward the most interesting dose level. Nevertheless, even if these stabilizers have no significant value, they deserve our interest on the mechanisms involved, including the induction of cytokine.

It has to be agreed that lipid A analogues, such as OM-174, do not appear very promising when used alone in cancer patients. Nevertheless our team has been working extensively in animal model and our knowledge of underlying mechanism or tumor growth inhibition led us to assess the possible synergy of OM-174 with different drugs. Like this, we have shown (unpublished data) that whereas treatment by either oxaliplatin or OM-174 alone doesn’t lead to regression of voluminous peritoneal tumors, induced in BDIX rats by intraperitoneal injection of syngeneic PROb colon cancer cells. Their combination induces the disappearance of carcinomatosis.

## Conclusion

We have shown that the lipid A analog, OM-174, administered twice weekly, for up to 15 injections and 1000 μg/m^2^ offers a predictable and overall favourable safety profile and good tolerability at biologically active concentrations. Clinical and biological activities deserve further experiments, likely in conjunction with chemotherapy, as preclinical experiments conducted in our unit suggest that this combination might be synergistic. To confirm this hypothesis, we have designed a phase Ib study in association with a derivate of platine that will soon be enrolling patients.

## Competing interest

All the authors except Jacques Bauer declare that they have no competing interests. Jacques Bauer is an employee of OM-Pharma which has been integrated in Vifor Pharma in 2009.

## Authors’ contributions

Conceived and designed: JB, JFJ, MB. Acquisition of data: NI, PF, SZ, MB. Analysis and interpretation of data: NI, PF, CP, KR, GL, JFJ, MB. Wrote the paper: NI, CP, JB, JFJ, MB. Read and approved the final version of the manuscript: NI, PF, CP, SZ, JB, KR, GL, JFJ, MB. All authors read and approved the final manuscript.

## Pre-publication history

The pre-publication history for this paper can be accessed here:

http://www.biomedcentral.com/1471-2407/13/172/prepub

## References

[B1] Becker N Altenburg HP Stegmaier C Ziegler H Report on trends of incidence (1970-2002) of and mortality (1952-2002) from cancer in Germany J Cancer Res Clin Oncol 2007 133 23 35 16896882 10.1007/s00432-006-0142-4 PMC12160771

[B2] Center MM Jemal A Ward E International trends in colorectal cancer incidence rates Cancer Epidemiol Biomarkers Prev 2009 18 1688 1694 10.1158/1055-9965.EPI-09-0090 19505900

[B3] Cress RD Morris C Ellison GL Goodman MT Secular changes in colorectal cancer incidence by subsite, stage at diagnosis, and race/ethnicity, 1992-2001 Cancer 2006 107 1142 1152 10.1002/cncr.22011 16835912

[B4] Edwards BK Ward E Kohler BA Eheman C Zauber AG Anderson RN Jemal A Schymura MJ Lansdorp-Vogelaar I Seeff LC, van BM, Goede SL, Ries LA: Annual report to the nation on the status of cancer, 1975-2006, featuring colorectal cancer trends and impact of interventions (risk factors, screening, and treatment) to reduce future rates Cancer 2010 116 544 573 10.1002/cncr.24760 19998273 PMC3619726

[B5] Karim-Kos HE De VE Soerjomataram I Lemmens V Siesling S Coebergh JW Recent trends of cancer in Europe: a combined approach of incidence, survival and mortality for 17 cancer sites since the 1990s Eur J Cancer 2008 44 1345 1389 10.1016/j.ejca.2007.12.015 18280139

[B6] Heron M Deaths: leading causes for 2007 Natl Vital Stat Rep 2011 59 1 95 21950210

[B7] Jemal A Siegel R Xu J Ward E Cancer statistics, 2010 CA Cancer J Clin 2010 60 277 300 10.3322/caac.20073 20610543

[B8] Evans C Dalgleish AG Kumar D Review article: immune suppression and colorectal cancer Aliment Pharmacol Ther 2006 24 1163 1177 10.1111/j.1365-2036.2006.03075.x 17014575

[B9] Kemp TJ Ludwig AT Earel JK Moore JM Vanoosten RL Moses B Leidal K Nauseef WM Griffith TS Neutrophil stimulation with Mycobacterium bovis bacillus Calmette-Guerin (BCG) results in the release of functional soluble TRAIL/Apo-2L Blood 2005 106 3474 3482 10.1182/blood-2005-03-1327 16037389 PMC1895062

[B10] Manegold C Gravenor D Woytowitz D Mezger J Hirsh V Albert G Al-Adhami M Readett D Krieg AM Leichman CG Randomized phase II trial of a toll-like receptor 9 agonist oligodeoxynucleotide, PF-3512676, in combination with first-line taxane plus platinum chemotherapy for advanced-stage non-small-cell lung cancer J Clin Oncol 2008 26 3979 3986 10.1200/JCO.2007.12.5807 18711188

[B11] Mauri D Kamposioras K Tsali L Bristianou M Valachis A Karathanasi I Georgiou C Polyzos NP Overall survival benefit for weekly vs. three-weekly taxanes regimens in advanced breast cancer: A meta-analysis Cancer Treat Rev 2010 36 69 74 10.1016/j.ctrv.2009.10.006 19945225

[B12] Keogh B Parker AE Toll-like receptors as targets for immune disorders Trends Pharmacol Sci 2011 32 435 442 10.1016/j.tips.2011.03.008 21529972

[B13] Cluff CW Monophosphoryl lipid A (MPL) as an adjuvant for anti-cancer vaccines: clinical results Adv Exp Med Biol 2009 667 111 123 10.1007/978-1-4419-1603-7_10 20665204

[B14] Palsson-McDermott EM O'Neill LA Signal transduction by the lipopolysaccharide receptor, Toll-like receptor-4 Immunology 2004 113 153 162 10.1111/j.1365-2567.2004.01976.x 15379975 PMC1782563

[B15] Inagawa H Nishizawa T Noguchi K Minamimura M Takagi K Goto S Soma G Mizuno D Anti-tumor effect of lipopolysaccharide by intradermal administration as a novel drug delivery system Anticancer Res 1997 17 2153 2158 9216680

[B16] Jeannin JF Onier N Lagadec P, von JN, Stutz P, Liehl E: Antitumor effect of synthetic derivatives of lipid A in an experimental model of colon cancer in the rat Gastroenterology 1991 101 726 733 1860636 10.1016/0016-5085(91)90532-p

[B17] Onier N Hilpert S Arnould L Saint-Giorgio V Davies JG Jeannin JF Jeannin JF Cure of colon cancer metastasis in rats with the new lipid A OM 174. Apoptosis of tumor cells and immunization of rats Clin Exp Metastasis 1999 17 299 306 10.1023/A:1006663017149 10545016

[B18] Parr I Wheeler E Alexander P Similarities of the anti-tumour actions of endotoxin, lipid A and double-stranded RNA Br J Cancer 1973 27 370 389 10.1038/bjc.1973.45 4713170 PMC2008799

[B19] Sassi N Paul C Martin A Bettaieb A Jeannin JF Jeannin J Lipid A induced response in vivo Lipid A in cancer therapy 2010 69 80 Springer edition 10.1007/978-1-4419-1603-7_7 20665201

[B20] Blondiau C Lagadec P Lejeune P Onier N Cavaillon JM Jeannin JF Correlation between the capacity to activate macrophages in vitro and the antitumor activity in vivo of lipopolysaccharides from different bacterial species Immunobiology 1994 190 243 254 10.1016/S0171-2985(11)80272-X 8088853

[B21] van de Wiel PA van der PA Bloksma N Role of tumour necrosis factor in the tumour-necrotizing activity of agents with diverging toxicity Cancer Immunol Immunother 1991 33 115 120 10.1007/BF01742539 2036659 PMC11038284

[B22] Reisser D Pance A Jeannin JF Mechanisms of the antitumoral effect of lipid A Bioessays 2002 24 284 289 10.1002/bies.10053 11891766

[B23] Onier N Lejeune P Martin M Hammann A Bauer J Hirt P Lagadec P Jeannin JF Involvement of T lymphocytes in curative effect of a new immunomodulator OM 163 on rat colon cancer metastases Eur J Cancer 1993 29A 2003 2009 8280496 10.1016/0959-8049(93)90462-o

[B24] Shimizu T Iida K Iwamoto Y Yanagihara Y Ryoyama K Asahara T Ikeda K Achiwa K Biological activities and antitumor effects of synthetic lipid A analog linked N-acylated serine Int J Immunopharmacol 1995 17 425 431 10.1016/0192-0561(95)00014-S 7591367

[B25] D'Agostini C Pica F Febbraro G Grelli S Chiavaroli C Garaci E Antitumour effect of OM-174 and cyclophosphamide on murine B16 melanoma in different experimental conditions Int Immunopharmacol 2005 5 1205 1212 10.1016/j.intimp.2005.02.013 15914325

[B26] Kiani A Tschiersch A Gaboriau E Otto F Seiz A Knopf HP Stutz P Farber L Haus U Galanos C Mertelsmann R Engelhardt R Downregulation of the proinflammatory cytokine response to endotoxin by pretreatment with the nontoxic lipid A analog SDZ MRL 953 in cancer patients Blood 1997 90 1673 1683 9269788

[B27] de Bono JS Dalgleish AG Carmichael J Diffley J Lofts FJ Fyffe D Ellard S Gordon RJ Brindley CJ Evans TR Phase I study of ONO-4007, a synthetic analogue of the lipid A moiety of bacterial lipopolysaccharide Clin Cancer Res 2000 6 397 405 10690516

[B28] Vosika GJ Barr C Gilbertson D Phase-I study of intravenous modified lipid A Cancer Immunol Immunother 1984 18 107 112 6391653 10.1007/BF00205743 PMC11039296

[B29] Rickham PP Human experimentation. Code of ethics of the World Medical Association. Declaration of Helsinki. Br Med J 1964 2 177 14150898 10.1136/bmj.2.5402.177 PMC1816102

[B30] Therasse P Arbuck SG Eisenhauer EA Wanders J Kaplan RS Rubinstein L Verweij J Van GM van Oosterom AT Christian MC Gwyther SG New guidelines to evaluate the response to treatment in solid tumors. European Organization for Research and Treatment of Cancer, National Cancer Institute of the United States, National Cancer Institute of Canada J Natl Cancer Inst 2000 92 205 216 10.1093/jnci/92.3.205 10655437

[B31] Lirussi F Rakotoniaina Z Madani S Goirand F Breuiller-Fouche M Leroy MJ Sagot P Morrison JJ Dumas M Bardou M ADRB3 adrenergic receptor is a key regulator of human myometrial apoptosis and inflammation during chorioamnionitis Biol Reprod 2008 78 497 505 10.1095/biolreprod.107.064444 17989355

[B32] Prunet C Montange T Vejux A Laubriet A Rohmer JF Riedinger JM Athias A Lemaire-Ewing S Neel D Petit JM Steinmetz E Brenot R Gambert P Lizard G Multiplexed flow cytometric analyses of pro- and anti-inflammatory cytokines in the culture media of oxysterol-treated human monocytic cells and in the sera of atherosclerotic patients Cytometry A 2006 69 359 373 16604541 10.1002/cyto.a.20272

[B33] Ducloux D Deschamps M Yannaraki M Ferrand C Bamoulid J Saas P Kazory A Chalopin JM Tiberghien P Relevance of Toll-like receptor-4 polymorphisms in renal transplantation Kidney Int 2005 67 2454 2461 10.1111/j.1523-1755.2005.00354.x 15882292

[B34] Ellegaard M Bastholt L Jakobsen A Donskov F Schmidt H Interleukin-2-induced fever in relation to objective tumor response and survival in patients with metastatic melanoma J Clin Oncol 2010 28 15s

[B35] Engelhardt R Mackensen A Galanos C Phase I trial of intravenously administered endotoxin (Salmonella abortus equi) in cancer patients Cancer Res 1991 51 2524 2530 2021932

[B36] Rockwell C Morison D Qureshi N Jeannin J Lipid-A mediated tolerance and cancer therapy. Lipid A in cancer therapy 2010 81 99 10.1007/978-1-4419-1603-7_8 PMC3965205 20665202

[B37] Garay RP Viens P Bauer J Normier G Bardou M Jeannin JF Chiavaroli C Cancer relapse under chemotherapy: why TLR2/4 receptor agonists can help Eur J Pharmacol 2007 563 1 17 10.1016/j.ejphar.2007.02.018 17383632

